# Data for Genetic Analysis Workshop (GAW) 15 Problem 2, genetic causes of rheumatoid arthritis and associated traits

**DOI:** 10.1186/1753-6561-1-s1-s3

**Published:** 2007-12-18

**Authors:** Christopher I Amos, Wei Vivien Chen, Elaine Remmers, Katherine A Siminovitch, Michael F Seldin, Lindsey A Criswell, Annette T Lee, Sally John, Neil D Shephard, Jane Worthington, Francois Cornelis, Robert M Plenge, Ann B Begovich, Thomas D Dyer, Daniel L Kastner, Peter K Gregersen

**Affiliations:** 1 Departments of Epidemiology and Biomathematics, University of Texas, M.D. Anderson Cancer Center, 1155 Pressler Street, Unit 1340, Houston, Texas 77030, USA; 2 Genetics and Genomics Branch, NIAMS, NIH, 9 Memorial Drive, MSC 0908, Building 9, Room 1W108, Bethesda, Maryland 20892, USA; 3 Mount Sinai Hospital, SLRI, Room 656A, 600 University Avenue, Toronto, Ontario M5G 1X5 Canada; 4 Rowe Program of Human Genetics, Department of Medicine, 4303 Tupper Hall, University of California – Davis, Davis, California 95616, USA; 5 The Rosalind Russell Medical Research Center for Arthritis, Department of Medicine, Division of Rheumatology, University of California at San Francisco, California 94122, USA; 6 Center for Genomics and Human Genetics, North Shore-Feinstein Medical Research Institute, 350 Community Drive, Manhasset, New York 11030, USA; 7 Cancer Research-UK, Institute for Cancer Studies, School of Medicine and Biomedical Sciences, University of Sheffield, Beech Hill Road, Sheffield, S10 2RX, UK; 8 Centre for Integrated Genomic Medical Research, Stopford Building, University of Manchester, Oxford Road, Manchester, M13 9PL, UK; 9 GenHotel-EA 3886, University Evry-Paris 7/AP-HP, Evry-Genopole, Cedex, France; 10 Broad Institute of MIT and Harvard, Brigham and Women's Hospital, Division of Rheumatology, 7 Cambridge Center, Cambridge, Massachusetts 02142, USA; 11 Celera Diagnostics, 1401 Harbor Bay Parkway, Alameda, California 94502, USA; 12 Department of Genetics, Southwest Foundation for Biomedical Research, 7620 Northwest Loop 410, San Antonio, Texas 78227, USA

## Abstract

For Genetic Analysis Workshop 15 Problem 2, we organized data from several ongoing studies designed to identify genetic and environmental risk factors for rheumatoid arthritis. Data were derived from the North American Rheumatoid Arthritis Consortium (NARAC), collaboration among Canadian researchers, the European Consortium on Rheumatoid Arthritis Families (ECRAF), and investigators from Manchester, England. All groups used a common standard for defining rheumatoid arthritis, but NARAC also further selected for a more severe phenotype in the probands. Genotyping and family structures for microsatellite-based linkage analysis were provided from all centers. In addition, all centers but ECRAF have genotyped families for linkage analysis using SNPs and these data were additionally provided. NARAC also had additional data from a dense genotyping analysis of a region of chromosome 18 and results from candidate gene studies, which were provided. Finally, smoking influences risk for rheumatoid arthritis, and data were provided from the NARAC study on this behavior as well as some additional phenotypes measuring severity. Several questions could be evaluated using the data that were provided. These include comparing linkage analysis using single-nucleotide polymorphisms versus microsatellites and identifying credible regions of linkage outside the HLA region on chromosome 6p13, which has been extensively documented; evaluating the joint effects of smoking with genetic factors; and identifying more homogenous subsets of families for whom genetic susceptibility might be stronger, so that linkage and association studies may be more efficiently conducted.

## Background

Rheumatoid arthritis (RA) is a complex disease with a moderately strong genetic component. The recurrence risk ratio for siblings is typically estimated at around six in Caucasians, but with a broad range of values, primarily because the prevalence in the population is not well characterized [1]. The prevalence also varies among populations, ranging from around 0.8% in Caucasians to 10% in some Native American groups, although it is not clear that this is always the same phenotype. RA appears to be rare in rural African populations. Generally females are at higher risk than males, with about a three to one preponderance of females to males. The mean age of disease onset is in the fifth decade, with considerable variability in age at presentation, including occasional presentation in the teenage years.

The HLA region on 6p21 has been implicated by numerous studies, and there is consistent evidence that DR alleles contribute to disease risk. The 'shared epitope' hypothesis was proposed by Gregersen et al. [2] to explain the organization of risk for rheumatoid arthritis from DR alleles. According to this hypothesis, individuals who share a QK/RRAA motif in positions 70 to 74 of the DR molecule show an increased risk for disease. The alleles that confer increased risk for rheumatoid arthritis include DRB1*0101, 0102, 0104, 0105, **0401**, **0404**, **0405**, **0408**, **0409**, 1001, 1402, and 1406, with highest risk alleles being bolded [3]. This model was not quite sufficient to explain risk according to DR types and a newer model utilizing data from positions 70 to 74 has been developed [4]. Aside from these main effects, there is also evidence for an interaction or haplotypic effects including the class I region and the central MHC, along with certain DR alleles, notably DR3 [5,6].

Specific autoantibodies are noted to co-occur with rheumatoid arthritis. Rheumatoid factor (RF) IgM is a measure of active disease correlated with erosive arthritic disease. However, a more newly identified autoantibody, anti-cyclic citrullinated peptide (anti-CCP), is more specific for the disease and is a better predictor of erosive outcome [7]. Elevations of anti-CCP have been noted to predict increased risk for development of rheumatoid arthritis [8]. The shared epitope alleles are strongly associated with the presence of anti-CCP antibodies, and this effect is modulated by HLA-DR3 [7]. Alleles at the *PTPN22 *locus have been shown to confer an increased risk for RA [9]. At least two alleles of *PTPN22 *have been implicated as causing increased risk for RA; with the R620W allele in rs2476601 (hCV16021387) conferring 1.7- to 1.9-fold increased risk to heterozygotes and higher risks to homozygous carriers. Increased risk was also noted for either hCV8689108 or hCV25762283 [10], with some indeterminacy because of linkage disequilibrium among these markers (and others in the region). These findings have further been confirmed by analysis of transmission of *PTPN22 *alleles to affected offspring in families [11].

Additional loci that have been implicated include *PADI4*, which encodes the enzyme catalyzing citrullination in macrophages (on chromosome 1p), intron 1 of *SLC22A4 *on chromosome 5q, *RUNX1 *(on chromosome 1q), and a locus on chromosome 17 possibly predisposing to psoriasis. Marker data for these other loci could not be obtained, but have generally not shown consistent increases in risk for Caucasian populations. The *CTLA4 *locus on chromosome 4p has been associated with mildly increased risk for rheumatoid arthritis [12].

Aside from identified genetic factors and sex, few environmental cofactors have yet been identified as affecting risk for rheumatoid arthritis. However, current smoking confers about a two-fold increased risk [13]. Klareskog et al. [14] recently showed that the risk from smoking for rheumatoid arthritis is particularly high among individuals who have a shared epitope allele and who also have elevated levels of anti-CCP. The biological basis for this rather complex interaction appears to reflect increased citrullination of peptides among smokers, and presentation of citrullinated peptides by shared epitope alleles.

The primary goal of the studies that were submitted for the Genetic Analysis Workshop 15 has been to identify genetic factors that predispose for rheumatoid arthritis. Four independent academic groups and one company have provided data for the workshop. In addition, given some previously identified evidence for effects of smoking on rheumatoid arthritis risk and difference in risk according to gender, there is considerable interest in identify gene × environment and gene × gene combinations that yield particularly high risks to individuals for rheumatoid arthritis.

## Methods

Data for the workshops were provided by five centers. Two centers (Canada and NARAC) had SNP genotyping performed jointly. The data were transmitted from each center to the University of Texas M.D. Anderson Cancer Center, where the data sets were checked to assure the availability of data definitions and to evaluate the formatting and completeness of the data transfer. Subsequently, data were transmitted to the Southwest Foundation for Biomedical Research for integration and transfer to GAW15 participants. Questions about data integrity or meaning were transmitted to the University of Texas M.D. Anderson Cancer Center, which then interacted with the data providers to obtain answers. All affected subjects in all of the studies met the standard ACR criteria for affection with rheumatoid arthritis [15]. A distribution of selected clinical characteristics among study participants is provided in Table 1.

**Table 1:** Distribution of selected clinical characteristics among study participants from rheumatoid arthritis studies for GAW15 Problem 2

Study source	US Micros^a^	US-SNPs^b^	Canada Affymetrix	Canada Illumina^c^	France	UK Cohort 1^d^	UK Cohort 2^e^
Ascertainment criteria	2 ACR+ sibs, 1 with erosions	2 ACR+ sibs, 1 with erosions	2 ACR+ sibs		2 ACR+ sibs	2 ACR+ sibs	2 ACR+ sibs

Number of families	511	757	79	60	88	174	195
Affected sibs/family	2.13	2.15	2	2	2.18	2.13	2.09
% SE+	71%	79%	N/A	N/A	N/A	90%	82%
% Erosion	95%	97%	39%	N/A	N/A	76%	71%
Median age onset	39.1	38.9	43.8	N/A	N/A	39.3	44.0
% RF+	81.1%	74.3%	71.1%	N/A	N/A	62.5%	56.7%

^a^Numbers from Jawaheer et al. [17]. One family was eliminated from GAW15 data because one sib did not meet ACR criteria for RA.

^b^Of these families, 9 had only phenotype information and no SNP genotypes, 509 families have both SNP and microsatellite data, 2 families have microsatellite data only, and 237 have SNP data only.

^c^47 families have genotype data using both Affymetrix and Illumina platforms.

^d^174 affected families had microsatellite data and of these 157 also had SNP genotyping.

^e^Selected microsatellite markers were genotyped for variable numbers of these families.

### The North American Rheumatoid Arthritis Consortium study (NARAC)

#### Microsatellite scans

The familial clustering patterns, association with extra-articular findings, and correlation in ages of onset in most of the NARAC collection have been described by Jawaheer et al. [16]. Affected subjects did not have other autoimmune diseases that include an arthritic component such as systemic lupus erythematosis, Crohn's disease, or psoriasis. NARAC has performed microsatellite scans [17] using the Applied Biosystems, Inc. (ABI) standard panels for 511 multiplex families that include 676 sib pairs (and parents were available) as well as a handful of somewhat larger families. About 90% of the families are Caucasian. The Kong and Cox LOD score for chromosome 6p is approximately 17 and extends rather centromerically, suggesting a possible second locus on 6p [18].

#### SNP scans

Illumina performed analysis of about 5600 genome-wide SNPs on all families including 66 families from Katherine Siminovitch, a collaborator in Canada. Results of the analysis of the NARAC Caucasian families were published and indicate previously unreported linkages on chromosomes 2, 4, and 11 along with the known linkage on chromosome 6p [18].

#### Association mapping

A dense panel of 2719 SNPs were genotyped by Illumina for an approximately 10-kb region of chromosome 18q that showed evidence for linkage in the U.S. and French linkage scans. Of these, 2300 met quality control criteria and have been retained and distributed for analysis. These markers were individually genotyped on 460 cases and 460 controls. Controls were recruited from a New York City population and cases have been recruited from multiple U.S. centers. As a part of the data release process, we also distributed the estimated Northern versus Southern European ancestry of cases and controls [19] because the European ancestry of cases and controls deviates, given different catchements for cases versus controls.

#### Quantitative phenotypes

Two quantitative phenotypes that are used for identifying RA-affected individuals include anti-CCP and RF-IgM. The heritability of these measures is hard to obtain from the selected sib pairs we are studying. After proband correction, the heritability estimates are 11% and 30%; before correction the heritabilities are 15% and 67%. Linkage analysis of Rf-IgM and anti-CCP phenotypes with microsattelites [20] showed strong evidence for linkages of these phenotypes to chromosome 6 and LOD scores over 1 for linkage of both phenotypes to 1p21.1, 5q15, 8p23.1, 16p12.1, 16q23.1, and 18q21.31.

#### Clinical measures

Clinical measures that are available include age at onset, sex, ethnicity, presence of erosions, and duration of disease. Data were also provided concerning the physician-reported severity of disease (JAM scores) as well as the patient's functional status (HAQ scores). Smoking increases risk for rheumatoid arthritis, and limited smoking data are available for families and controls. Digitized hand radiographs are available on the NARAC website. The currently available X-ray scores were derived by a single radiologist at the Bethesda Naval Medical Center. Jawaheer et al. studied clinical characteristics of the study subjects [13].

### The European Consortium on Rheumatoid Arthritis Families (ECRAF)

ECRAF provided high-density microsatellite data from 88 families, including 75 affected sib pairs, 12 affected sib trios, and 1 affected sib quaternion typed with 1089 microsatellite markers [21]. *PTPN22 *genotypes are available from this collection. All affected subjects from this study met ACR criteria [16].

#### United Kingdom – Manchester

The UK group led by Jane Worthington and Sally John provided data from analysis of 10,156 SNP markers that were genotyped and passed quality control filters on 157 families [22]. In addition microsatellite data from an entire genome-wide screen was available from 369 markers that were genotyped on 174 families (screen 1) with two or more affected siblings and from 10 candidate regions that showed evidence in screen 1 for linkage genotyped for 89 markers on a different set of 195 families with two or more affected siblings [23]. All affected individuals have been classified as affected according to ACR criteria [16].

#### Canada – Toronto

The Canadian group, led by Katherine Siminovitch, provided 60 families that have been genotyped using the Illumina platform used by NARAC (performed at the same time as the NARAC study) as well as 79 families (one sib pair had only one affected sibling and is excluded from tabulations) that were genotyped using an Affymetrix 100 K platform. Patients (*n *= 86) were recruited from large clinical populations in the Toronto area in collaboration with academic-based rheumatologists. Sibships with affected pairs were also recruited from academic centers in Nova Scotia (*n *= 72). All affected patients met 1987 revised criteria for RA [16]. The presence of other diseases that are accompanied by inflammatory arthritis, such as psoriasis or inflammatory bowel disease, was an exclusionary criterion for families. Informed consent was obtained from every subject, including all participating family members, and approval of the local institutional review board was secured at every recruitment site prior to enrollment. Of the families studied, 76 were European Caucasian, 1 was Indian, 1 was South-East Asian, and 1 was Ashkenazic. The median time to onset with RA was 42 years of age, 25% of cases were male, and 37% had erosions.

## Discussion

Although previous research has identified a few loci that consistently show association with rheumatoid arthritis, a great deal remains unknown about the mechanisms by which genetic factors interrelate to increase disease risk, and the impact that environmental factors such as smoking behavior have upon disease risk. The collaborative approach that has been adopted by rheumatoid arthritis researchers provided an excellent platform for integrating data from multiple sites in an effort to obtain a larger and more powerful collection of data resources than was possible from a single site. In addition, the Genetic Analysis Workshop platform allowed the data to be more thoroughly and impartially queried than is possible by any of the single collaborating sites. The following sets of questions were posed to the GAW participants:

1. When analyzing dense SNP data and when parental data are missing what is the best procedure for dealing with linkage disequilibrium? We have noted some very high LOD scores in both the Canadian and NARAC data sets that can be eliminated by removing excess LD, but does this lead to an excess loss of information?

2. How best to analyze data from the pseudo-autosomal region?

3. Is there evidence for gene × environment interactions? Do *PTPN22 *and the shared epitope interact with smoking behavior to increase risk for disease? Can subgroups with very high risk for disease be identified? (Note this may not be the best data set to answer this question.) Does smoking influence severity or age to onset of disease? What are the best procedures for using known covariates such as sex, anti-CCP levels, and shared epitope status to identify genetic loci influencing disease susceptibility?

4. Do the quantitative variables provide any increased power to identify genetic loci? Although microsatellite data have been analyzed for the quantitative traits, at this time the SNP data have not yet been analyzed.

5. Meta-analysis: What are the best ways to combine data across the studies? Is there any strong evidence for gene × gene interactions? Is there more than one locus on chromosome 6 influencing disease risk? Can the *PTPN22 *locus on chromosome 1 be identified by linkage?

6. Association data: Are there any loci on chromosome 18 that reliably predict disease risk? Are there any subsets with particularly high risks for disease?

## Conclusion

The data that were provided is composed largely of affected sib-pairs. Efforts were made to collect extended relatives when they were available. However, the aggregation of rheumatoid arthritis in families usually occurs in siblings and parents of the proband and only rarely occurs in extended pedigrees. Due to fiscal constraints, only a few of the families from NARAC included unaffected relatives, and none of the other sites provided data from unaffected relatives. A variety of methods are required to unravel the complex genetic and environmental interactions that cause this complex disease. The value of the genetic analysis workshop has been that it brings together analysts with a wide variety of skills and approaches. The data providers were thankful for the opportunity to have the extensive data that have been developed and studied in detail by a wide range of analysts.

## Competing interests

The authors report the following competing interests relating to this publication. Dr. Lindsey Criswell reports receiving consulting fees from Celera Diagnostics. Dr Ann Begovich is an employee of Celera which funded some of the genotyping work included in this manuscript.
